# Sex-specific gray matter underpinnings of anxiety and their mediating mechanisms in *de novo* Parkinson’s disease

**DOI:** 10.3389/fnagi.2026.1769488

**Published:** 2026-04-13

**Authors:** Guixiang He, Xiaofang Huang, Yi Xing, Lingling Yang, Haicun Shi, Haihua Sun, Weiguo Liu, Yuanzhang Zhou, Pinglei Pan

**Affiliations:** 1 Department of Neurology, Affiliated Hospital 6 of Nantong University, Yancheng Third People's Hospital, Yancheng, China; 2 Department of Neurology, Affiliated Nanjing Brain Hospital, Nanjing Medical University, Nanjing, China; 3 Department of Immunology, School of Medicine and Holistic Integrative Medicine, Nanjing University of Chinese Medicine, Nanjing, China

**Keywords:** anxiety, *de novo*, mediation analyses, Parkinson’s disease, sex differences, voxel-based morphometry

## Abstract

**Objective:**

Anxiety is a prevalent non-motor symptom in Parkinson’s disease (PD) with observed sex differences. This study aimed to investigate sex-specific gray matter volume (GMV) associations with anxiety and preliminarily explore the potential mediating role of these regional GMV alterations in the relationship between depressive and anxiety symptoms in *de novo* PD patients.

**Methods:**

A total of 108 *de novo* PD patients were enrolled. Anxiety and depressive symptoms were assessed using the Hamilton Anxiety Rating Scale (HAMA) and the 24-item Hamilton Depression Rating Scale (HAMD-24), respectively. Whole-brain GMV was analyzed using voxel-based morphometry (VBM) on T1-weighted MRI data. A 2 × 2 analysis of variance (sex × anxiety status) was conducted to examine main and interaction effects, controlling for age, education, HAMD-24 total score, and total intracranial volume. Sex-stratified, cross-sectional mediation analyses were subsequently performed.

**Results:**

Whole-brain analysis revealed a significant sex-by-anxiety interaction in the right precuneus (*p* = 0.015). Post-hoc correlation analyses indicated that anxiety severity was negatively associated with GMV in the right precuneus in male patients (*r* = −0.387, *p* = 0.010) and with GMV in the left insula in female patients (*r* = −0.437, *p* = 0.004). Mediation analyses suggested that right precuneus GMV partially mediated the association between HAMD-24 and HAMA scores in males (mediation proportion: 17.1%). Similarly, left insular GMV partially mediated this association in females (mediation proportion: 15.4%).

**Conclusion:**

This study identified sex-specific GMV correlates of anxiety in *de novo* PD, primarily involving the precuneus in males and the insula in females. These structural differences may partially underlie the co-occurrence of depressive and anxiety symptoms, highlighting sexually dimorphic neuroanatomical substrates for affective disturbances in PD.

## 1 Introduction

Anxiety is a prevalent non-motor symptom in Parkinson’s disease (PD), which not only significantly impairs patients’ health-related quality of life, but also is closely associated with accelerated motor function progression, higher fall risk, and reduced treatment adherence (Broen et al., 2016; Goltz et al., 2024; Lonka et al., 2025; Zhao et al., 2025a). Epidemiological studies indicate that anxiety affects approximately 45% of PD patients (Mammen et al., 2025), with a higher prevalence reported in females than in males (64% vs. 55%; *p* < 0.01) (Tropea et al., 2024). While PD-related anxiety can be managed with pharmacotherapy or cognitive-behavioral therapy (Pontone and Mills, 2021; van Wegen et al., 2024), these approaches are not specific to PD and fail to account for sex differences, often resulting in inconsistent treatment outcomes. It is therefore necessary to elucidate sex-specific neural mechanisms to provide a basis for developing precision intervention strategies. Although sex differences in motor phenotypes are well established (Meoni et al., 2020; Cattaneo and Pagonabarraga, 2025), the sex-specific neuroanatomical substrates of anxiety in PD remain poorly elucidated. Clarifying this mechanism can provide important theoretical reference and preliminary research basis for the differential diagnosis of PD-related anxiety, the development of sex-specific personalized intervention strategies, and the screening of imaging biomarkers for evaluating the efficacy of anti-anxiety treatment.

Sex differences in anxiety are not unique to PD but have been widely documented in the general population (Bangasser and Cuarenta, 2021). These prior studies have consistently confirmed a female predominance in anxiety prevalence, and key brain regions regulating emotional processing and anxiety (including the amygdala, prefrontal cortex, insula, and precuneus—the core regions of interest in this study) exhibit well-documented sex-specific structural and functional modulation (Cahill et al., 2004; Lopez-Larson et al., 2011; Tan et al., 2020; Shi et al., 2023). Gonadal hormones, via their effects on sex steroid receptor genes (ESR1, ESR2, AR), are established as the core biological mediators of these sex-related neuroanatomical and functional differences (Nakajima et al., 2014; Tan et al., 2020; Rigney et al., 2021; Nguyen et al., 2025). Despite the well-characterized sex differences in anxiety-related gray matter structure in the general population, sex-specific gray matter morphological correlates of anxiety in PD patients remain largely under investigated. In the present study, we employed voxel-based morphometry (VBM) to explore anxiety-related alterations in gray matter volume (GMV) between male and female PD patients, aiming to elucidate the neuroanatomical basis underlying sex differences in anxiety within the PD population.

Existing neuroimaging evidence has been systematically synthesized to demonstrate that the fear circuit and the limbic cortico-striato-thalamocortical circuit are critically involved in PD-related anxiety (Carey et al., 2021), with well-documented structural and neurochemical alterations within these networks (Volkmann et al., 2010; Vriend et al., 2016; Thobois et al., 2017; Zhang et al., 2022; Wang et al., 2023). The core neuropathology of PD, including dopaminergic degeneration and *α*-synuclein spread, is widely posited to disrupt these key affective circuits (McGregor and Nelson, 2019; Mohebi et al., 2019; Douma and de Kloet, 2020; Liu et al., 2020; Nguyen et al., 2021). This mechanistic framework is further supported by positron emission tomography (PET) studies, which have linked the severity of PD-related anxiety to reduced serotonergic and dopaminergic function in limbic regions, highlighting the critical role of neurotransmitter interactions in this condition (Weintraub et al., 2005; Moriyama et al., 2011; Erro et al., 2012; Maillet et al., 2016; Wang et al., 2017; Joling et al., 2018).

However, several critical gaps remain to be addressed in existing research. First, interpreting these findings is complicated by the modulatory effects of chronic dopaminergic medication on brain structure and function (Lee et al., 2025; Wang et al., 2025), as most studies have enrolled patients receiving long-term dopaminergic treatment, making it difficult to isolate disease-specific neuroanatomical alterations. Second, existing studies have not uniformly applied strict inclusion criteria to exclude patients with pre-existing primary anxiety disorder before the onset of PD motor symptoms, which makes it hard to fully distinguish whether the observed neural alterations are specific to PD pathology or inherent characteristics of primary anxiety. Third, existing neuroimaging studies have largely overlooked the potential sex-dependent expression of anxiety-related neural correlates (McEwen et al., 2016), and few have elucidated the role of sex-specific brain region alterations in the comorbidity of anxiety and depression in PD, which hinders the development of sex-informed clinical interventions for PD-related anxiety.

To address these gaps, we conducted a whole-brain VBM analysis in 108 *de novo* PD patients, with strict exclusion of patients with pre-existing primary anxiety disorder prior to the onset of PD motor symptoms. We hypothesized that: (1) anxiety in *de novo* PD may be associated with regional GMV alterations; (2) these neural correlates may exhibit significant sexual dimorphism, potentially implicating distinct large-scale brain networks; and (3) sex-specific GMV alterations may play a mediating role in the association between depressive symptoms and anxiety symptoms in PD patients. The potential implications of these findings for understanding the pathophysiology of PD-related anxiety and the development of sex-informed clinical strategies are discussed in detail below.

## 2 Materials and methods

### 2.1 Participants

This study focuses on the interaction between anxiety and sex and the associated neuroanatomical basis within the PD patient population. Therefore, a subgroup analysis design of PD patients was adopted, and no healthy control group was included. A total of 108 right-handed, *de novo* PD patients (aged 45–75 years) were retrospectively included from the Department of Neurology at our institution between October 2018 and October 2022. All patients met the 2015 International Parkinson and Movement Disorder Society (MDS) Clinical Diagnostic Criteria for PD (Postuma et al., 2015) and had no history of treatment with antiparkinsonian, antidepressant, or anxiolytic medications. *De novo* PD was defined as patients with a first diagnosis of idiopathic PD who had not received any antiparkinsonian, antidepressant, or anxiolytic treatment prior to enrollment. Eligible participants without MRI contraindications underwent 3 T structural MRI and comprehensive neuropsychological assessment.

Exclusion criteria were: (1) significant structural brain abnormalities (e.g., cerebrovascular disease, epilepsy, or prior neurosurgery); (2) secondary or atypical parkinsonism (e.g., drug-induced parkinsonism, multiple system atrophy, or progressive supranuclear palsy); (3) excessive head motion during MRI (translation > 2 mm or rotation > 2°); (4) comorbid major neuropsychiatric disorders (e.g., schizophrenia, bipolar disorder, or other neurodegenerative diseases); or (5) pre-existing primary anxiety or depressive disorders prior to the onset of PD motor symptoms.

### 2.2 Clinical assessment

All patients completed a standardized protocol including demographic data collection (age, sex, disease duration, education, occupation, residential environment), 3 T MRI, and neuropsychological evaluation. Global cognitive function was screened using the Chinese version of the Mini-Mental State Examination (MMSE), with the application of education-adjusted cutoff scores (Li et al., 2016). Anxiety and depressive symptoms were evaluated using the Hamilton Anxiety Rating Scale (Hamilton, 1959) (HAMA) and the 24-item Hamilton Depression Rating Scale (Hamilton, 1980) (HAMD-24), respectively. Sleep quality was measured with the Parkinson’s Disease Sleep Scale (Chaudhuri et al., 2002) (PDSS). Motor symptoms were rated with Parts II (activities of daily living) and III (motor examination) of the Unified Parkinson’s Disease Rating Scale (UPDRS-II and UPDRS-III) (Jankovic et al., 1990), and disease severity was staged using the modified Hoehn and Yahr (H-Y) scale (Goetz et al., 2004).

Based on established criteria for anxiety in PD, patients were classified into anxious (A-PD) and non-anxious (NA-PD) subgroups using a HAMA cutoff score of ≥11 (Leentjens et al., 2011), enabling sex-specific analysis of anxiety-related characteristics.

### 2.3 MRI data acquisition

All participants were scanned using a 3.0 T Siemens Verio system (Siemens Healthineers, Erlangen, Germany) with an 8-channel phased-array head coil at the Affiliated Brain Hospital of Nanjing Medical University. Participants were positioned supine, and foam padding was used to minimize head motion. Structural MRI data were acquired using a 3D T1-weighted magnetization-prepared rapid gradient-echo (MPRAGE) sequence. The acquisition parameters were: repetition time/echo time/inversion time (TR/TE/TI) = 2530/3.34/1100 ms, flip angle = 7°, field of view (FOV) = 256 × 256 mm^2^, and acquisition matrix = 256 × 192 × 128. This resulted in a volumetric dataset with a nominal voxel size of 1.0 × 1.33 × 1.33 mm^3^. The acquisition time was 8 min.

### 2.4 VBM preprocessing

The rationale for selecting GMV as the primary research indicator is as follows: (1) GMV provides a direct *in vivo* measure of the structural integrity of cerebral gray matter, a core readout for neuroanatomical alterations in neurodegenerative diseases including PD (Ashburner and Friston, 2000; Xu et al., 2020); (2) We quantified GMV using VBM, a well-established, widely validated approach for PD neuroanatomical research that offers unbiased whole-brain coverage and highly reproducible quantifiable results (Vanasse et al., 2018); (3) Compared with functional imaging metrics, which are vulnerable to transient physiological states, GMV has superior temporal stability, making it ideal for identifying trait-related neuroanatomical changes linked to chronic anxiety in our *de novo* PD cohort (Honey et al., 2007; Wang et al., 2025).

T1-weighted structural images were preprocessed using VBM implemented in the Computational Anatomy Toolbox (CAT12; http://dbm.neuro.uni-jena.de/cat12/) within Statistical Parametric Mapping (SPM12; http://www.fil.ion.ucl.ac.uk/spm/). Preprocessing included quality control (visual inspection for artifacts, manual reorientation to the anterior commissure), tissue segmentation into gray matter (GM), white matter (WM), and cerebrospinal fluid (CSF) using the “New Segment” algorithm and DARTEL registration (Ashburner, 2007). The resulting GM segments were spatially normalized to the Montreal Neurological Institute (MNI) space at 2 × 2 × 2 mm^3^ resolution and smoothed with an 8-mm full-width-at-half-maximum (FWHM) Gaussian kernel to enhance signal-to-noise ratio and mitigate inter-subject anatomical variability. Total intracranial volume (TIV) was estimated and included as a covariate in subsequent group-level analyses.

### 2.5 Statistical analysis

Statistical analyses were performed using IBM SPSS Statistics (version 25.0) and SPM12. Continuous variables are presented as mean ± standard deviation, ordinal variables as median (interquartile range), and categorical variables as frequency (percentage). Group comparisons (based on sex and anxiety status) for demographic and clinical variables were conducted using two-way ANOVA, Mann–Whitney U, or chi-square/Fisher’s exact tests, as appropriate, with Bonferroni post-hoc correction where applicable.

Whole-brain VBM was performed in SPM12 using a full factorial two-way ANOVA (factors: sex and anxiety status), controlling for age, education, HAMD-24 score, and total intracranial volume. Results were thresholded at a voxel-level of *p* < 0.001 and cluster-level family-wise error (FWE) correction of *p* < 0.05 (Gaussian random field theory), with an additional cluster-extent threshold of 50 voxels. Post-hoc region-of-interest (ROI) analyses on significant clusters used Bonferroni-corrected t-tests. Partial correlations (controlling for age, education, and HAMD-24) assessed relationships between ROI GMV and anxiety severity (HAMA scores) within each sex. In addition, partial correlation analyses were performed to assess the correlations between ROI GMV and UPDRS-III score and modified H-Y stage, respectively, among all 108 PD patients and 56 non-anxious PD patients, with age and education level controlled for, to clarify the association between GMV alterations and PD itself.

To identify predictors of anxiety, sex-stratified linear regression analyses were performed. Variables showing a univariate association with HAMA scores (*p* < 0.05) were entered into stepwise multivariable models. Finally, to test whether sex-specific GMV mediated the depression-anxiety relationship, separate mediation analyses were conducted using the bias-corrected bootstrap method (5,000 resamples). In these models, HAMD score was the independent variable, HAMA score the dependent variable, and the GMV of the key sex-specific region (right precuneus in males, left insula in females) the mediator, with age, education, and intracranial volume as covariates.

## 3 Results

### 3.1 Sociodemographic and clinical characteristics

The study included 108 *de novo* PD patients, categorized into four groups based on sex and anxiety status: anxious males (A-PD, *n* = 25), anxious females (A-PD, *n* = 27), non-anxious males (NA-PD, *n* = 32), and non-anxious females (NA-PD, *n* = 24). Demographic and clinical characteristics are summarized separately in Tables 1 (sociodemographic) and 2 (clinical measures).

**Table 1:** Demographic characteristics of *de novo* A-PD and NA-PD males and females.

Variable	A-PD Male (*n* = 25)	A-PD Female (*n* = 27)	NA-PD Male (*n* = 32)	NA-PD Female (*n* = 24)	Group effect test stat (*p*-value)	Sex effect test stat (*p*-value)
Age (years)	60.94 ± 5.99 (52–74)	60.15 ± 6.64 (45–72)	56.53 ± 9.76 (45–72)	60.24 ± 7.83 (45–73)	1.267 (0.263)	0.456 (0.501)
Disease duration (years)	1.75 ± 1.25 (0.5–6)	2.06 ± 1.83 (0–6)	1.49 ± 1.27 (0.5–7)	1.37 ± 0.96 (0.2–3)	2.581 (0.112)	0.082 (0.776)
Education (years)	11.22 ± 4.68 (0–19)	9.93 ± 2.74 (5–16)	10.72 ± 2.81 (3–15)	10.19 ± 2.77 (6–16)	0.067 (0.796)	1.667 (0.200)
Occupation (*n*, %)						
Physical labor	7 (28.00)	9 (33.35)	12 (37.50)	6 (25.00)	0.024 (0.878)	0.192 (0.661)
Mental labor	18 (72.00)	18 (66.67)	20 (62.50)	18 (75.00)		
Residential environment (*n*, %)						
Rural	5 (20.00)	6 (22.22)	10 (31.25)	5 (20.83)	0.468 (0.494)	0.332 (0.565)
Urban	20 (80.00)	21 (77.78)	22 (68.75)	19 (79.17)		

Data are mean ± SD (range) or frequency (%). Between-group comparisons were performed using two-way ANOVA (for continuous variables) or chi-square/Fisher’s exact tests (for categorical variables). No significant main or interaction effects were found for sex or anxiety status (all *p* > 0.05).

A-PD, Parkinson’s disease with anxiety; NA-PD, Parkinson’s disease without anxiety.

Groups were well-matched on all demographic variables (all *p* > 0.05; Table 1). As expected, A-PD patients demonstrated significantly greater clinical severity than NA-PD patients across multiple domains, including higher depressive symptoms, more severe motor impairment, reduced daily living function, and poorer sleep quality (all *p* < 0.005; Table 2). No significant sex-by-anxiety interactions were found for these clinical measures. A strong main effect of sex was observed for estimated intracranial volume (eTIV), with males having larger volumes than females (*p* < 0.001).

**Table 2:** Clinical characteristics of *de novo* A-PD and NA-PD males and females.

Variable	A-PD male (*n* = 25)	A-PD female (*n* = 27)	NA-PD male (*n* = 32)	NA-PD female (*n* = 24)	Group effect test stat (*p*-value)	Sex effect test stat (*p*-value)
MMSE scores	27.33 ± 2.45 (25–30)	26.30 ± 2.77 (24–30)	27.84 ± 2.73 (25–30)	28.05 ± 1.77 (26–30)	−1.328 (0.723)	−1.562 (0.118)
HAMD scores	19.22 ± 9.72 (2–42)	18.85 ± 7.15 (6–41)	3.34 ± 2.74 (0–12)	3.67 ± 1.80 (0–7)	160.471(<0.001)^a^	0.003 (0.960)
PDSS scores	109.28 ± 25.92 (60–145)	112.44 ± 21.23 (57–143)	136.63 ± 15.63 (76–150)	141.57 ± 5.58 (132–150)	54.700(<0.001)^a^	1.164 (0.283)
UPDRS-II score	9.56 ± 4.77 (3–17)	9.04 ± 4.42 (2–19)	6.63 ± 3.20 (0–15)	5.33 ± 3.31 (1–16)	16.073(<0.001)^a^	1.531 (0.219)
UPDRS-III scores	24.94 ± 11.70 (7–45)	24.33 ± 10.97 (5–54)	18.47 ± 8.44 (3–35)	18.05 ± 9.39 (5–53)	8.910(<0.001)^a^	0.114 (0.736)
Modified H-Y stage	2.00 (1.00, 2.12)	2.00 (1.50, 2.50)	1.50 (1.00, 2.00)	1.00 (1.00, 2.00)	−3.836 (<0.005)^a^	−0.741 (0.459)
Motor subtype (*n*, %)						
TD	13 (52.00)	12 (44.44)	16 (50.00)	9 (37.50)	2.101 (0.350)	1.113 (0.573)
Intermediate	2 (8.00)	3 (11.11)	1 (3.13)	1 (4.16)		
PIGD	10 (40.00)	12 (44.44)	15 (46.88)	14 (58.33)		
eTIV, mL	1572.59 ± 124.48	1353.16 ± 86.43	1527.17 ± 109.39	1379.08 ± 76.66	0.219 (0.641)	77.966(<0.001)^b^

Data are presented as mean ± standard deviation (range) for normally distributed continuous variables, median (interquartile range) for ordinal variables (modified H-Y stage), and frequency (percentage) for categorical variables. Group comparisons were conducted using two-way ANOVA (with Bonferroni post-hoc tests), Mann–Whitney U tests (for MMSE and H-Y stage), or chi-square tests.

A-PD, PD with anxiety; NA-PD, PD without anxiety; MMSE, Mini-Mental State Examination; HAMD, Hamilton Depression Rating Scale; PDSS, Parkinson’s Disease Sleep Scale; UPDRS, Unified Parkinson’s Disease Rating Scale; H-Y stage, Hoehn and Yahr stage; TD, Tremor-dominant; PIGD, Postural instability/gait difficulty; eTIV, estimated total intracranial volume.

a Significant anxiety-related differences (A-PD vs. NA-PD) in both sexes.

b Significant sex differences detected in both anxiety and non-anxiety groups.

### 3.2 Whole-brain VBM: main and interaction effects

Whole-brain analysis identified distinct neural patterns associated with sex and anxiety (Table 3). A significant main effect of anxiety was localized to the right middle temporal gyrus (*p* = 0.001). Main effects of sex were found in the bilateral insulae and left anterior cingulate cortex (ACC) (all *p* < 0.001). Critically, a significant sex-by-anxiety interaction was observed in the right precuneus (*p* = 0.015).

**Table 3:** GMV interaction and main effects of sex and anxiety in *de novo* PD (VBM).

Brain region (ALL)	Cluster size	*F*	Peak MNI coordinate		
			*X*	*Y*	*Z*
Interaction effect					
Precuneus_R	152	8.078	6	−49.5	70.5
Main effect of sex					
Insula_R	267	36.299	45	−4.5	−6
Insula_L	252	25.345	−42	−6	−6
Cingulum_Ant_L	258	26.877	−3	19.5	25.5
Main effect of anxiety					
Temporal_Mid_R	576	10.805	52.5	−54	12

Whole-brain two-way ANOVA (sex × anxiety status) with covariates (age, education, HAMD score, TIV). Thresholds: voxel-level *p* < 0.001, cluster-level *p* < 0.05, Gaussian random field corrected. Cluster size reported in number of voxels (2 × 2 × 2 mm^3^).

GMV, gray matter volume; ANOVA, analysis of variance; MNI, Montreal Neuroscience Institute; HAMD: Hamilton Depression Rating Scale; eTIV, estimated total intracranial volume; L, left; R, right.

### 3.3 Precuneus interaction and clinical correlations

**Figure 1:**
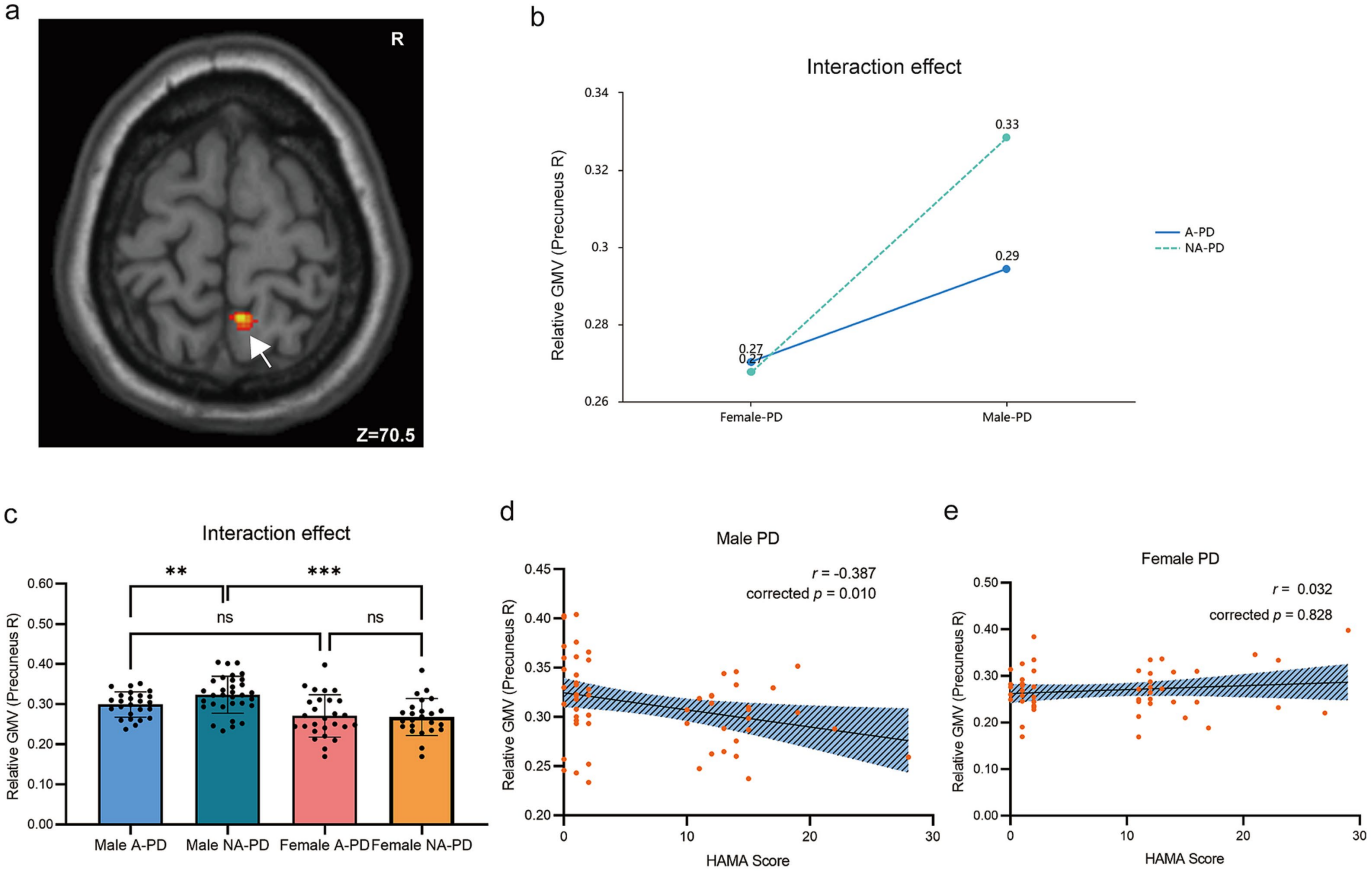
Sex-by-anxiety interaction effect on right precuneus GMV in *de novo* PD. **(a)** Brain cluster showing a significant sex × anxiety interaction (MNI: 6, −49.5, 70.5). **(b)** Line plot illustrating the interaction effect on GMV. **(c)** Post-hoc comparisons reveal reduced GMV in A-PD males compared to NA-PD males. **(d,e)** Partial correlation between right precuneus GMV and HAMA scores, stratified by sex (controlling for age, education, and HAMD scores). ^*^p < 0.05; ^**^p < 0.01; ^***^p < 0.001; ns, not significant. A-PD, Parkinson’s disease with anxiety; NA-PD, Parkinson’s disease without anxiety; GMV, Gray matter volume.

*Post-hoc* analysis of the right precuneus interaction showed a sexually divergent pattern (Figure 1). Anxious males had lower GMV than non-anxious males (*t* = −2.899, corrected *p* = 0.012), while no such difference was seen in females. Furthermore, lower precuneus GMV correlated with higher anxiety (HAMA scores) in males (*r* = −0.387, corrected *p* = 0.010) but not in females.

### 3.4 Right middle temporal gyrus shows an anxiety main effect across sexes

**Figure 2:**
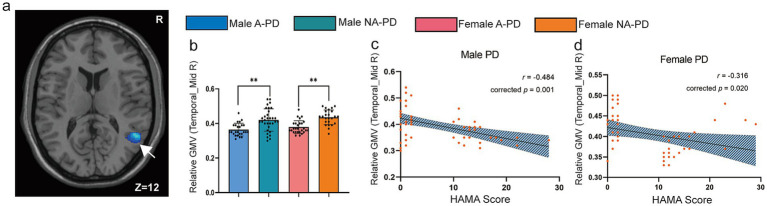
Anxiety-related main effect in the right MTG and correlation with symptom severity in *de novo* PD. **(a)** Cluster showing a significant anxiety main effect in the right MTG (peak MNI: 52.5, −54, 12). **(b)** Between-group comparisons (stratified by sex) showing lower MTG GMV in A-PD versus NA-PD patients. **(c,d)** Partial correlation scatter plots between right MTG GMV and HAMA scores (controlled for age, education, and HAMD-24 score) in males and females. *p < 0.05; **p < 0.01. A-PD, Parkinson’s disease with anxiety; NA-PD, Parkinson’s disease without anxiety; PD, Parkinson’s disease; GMV, gray matter volume; HAMA, Hamilton Anxiety Rating Scale; HAMD-24, 24-item Hamilton Depression Rating Scale; MNI, Montreal Neurological Institute; MTG, middle temporal gyrus.

A significant anxiety-related GMV reduction in the right middle temporal gyrus was found in both sexes (*p* = 0.001; Figure 2a). A-PD patients had lower GMV than NA-PD patients in this region (males: corrected *p* = 0.007; females: corrected *p* = 0.004; Figure 2b). GMV in this cluster negatively correlated with HAMA scores in both males (*r* = −0.484, corrected *p* = 0.001) and females (*r* = −0.316, corrected *p* = 0.020; Figures 2c,d).

### 3.5 Sex-specific insular correlates of anxiety

**Figure 3:**
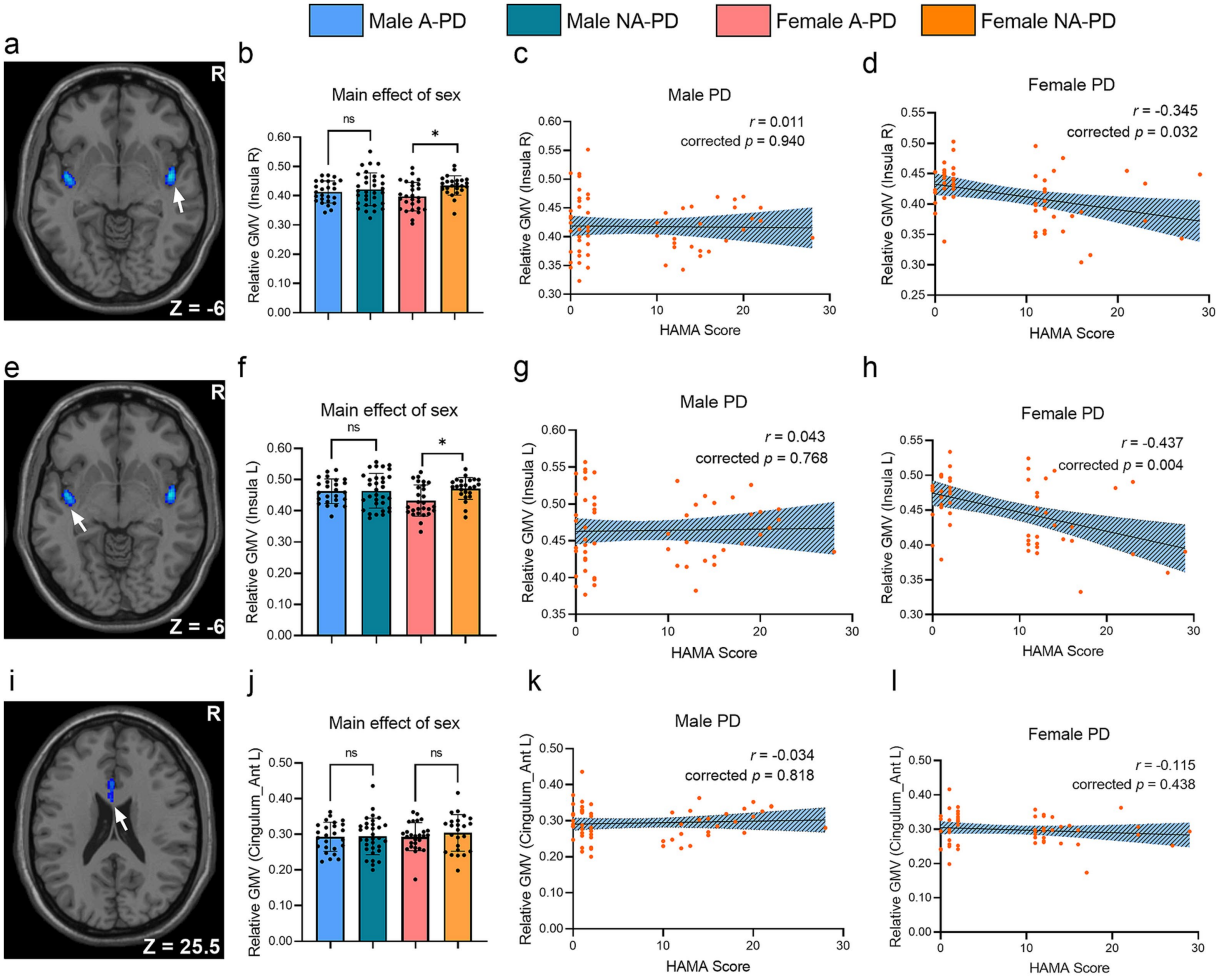
Sex-specific gray matter alterations associated with anxiety in *de novo* PD. **(a,e,i)** Brain regions showing main effects of sex: right insula, left insula, and left ACC. **(b,f,j)** Group differences in GMV between A-PD and NA-PD patients within each sex. **(c,d,g,h,k,l)** Partial correlations between regional GMV and HAMA scores, controlling for age, education, and HAMD scores. ^*^p < 0.05; ^**^p < 0.01; ^***^p < 0.001; ns, not significant. A-PD, Parkinson’s disease with anxiety; NA-PD, Parkinson’s disease without anxiety; GMV, gray matter volume; ACC, anterior cingulate cortex.

To examine anxiety-specific effects within each sex, we compared insular GMV between A-PD and NA-PD groups (Figure 3). Anxious females showed significant GMV reductions in the bilateral insulae compared to non-anxious females (right: *t* = −2.596, corrected *p* = 0.022; left: *t* = −2.421, corrected *p* = 0.034). No such anxiety-related differences were found in males.

Consistent with this, anxiety severity (HAMA scores) was negatively correlated with GMV in the left (*r* = −0.437, corrected *p* = 0.004) and right (*r* = −0.345, corrected *p* = 0.032) insula in females, but not in males.

### 3.6 Clinical correlation analysis

In the total PD population, partial correlation analyses (controlling for age, education, and HAMD-24 score) demonstrated no significant correlations between GMV in the right precuneus, left insula and right insula and UPDRS-III scores (*r* = −0.160, −0.195, −0.216, all *p* > 0.05) or modified H-Y stage (*r* = −0.113, −0.153, −0.174, all *p* > 0.05). Similarly, in the non-anxious PD subgroup, partial correlation analyses (controlling for age, education, and HAMD-24 score) still revealed no significant associations between GMV in the above brain regions and UPDRS-III scores (*r* = −0.145, −0.177, −0.262, all *p* > 0.05) or modified H-Y stage (*r* = −0.030, −0.077, −0.010, all *p* > 0.05). These results confirmed that the GMV alterations in the above key brain regions were not related to the motor severity or disease stage of PD itself, supporting the specificity of these GMV changes for PD-related anxiety.

### 3.7 Sex-specific predictors of anxiety severity

To identify the determinants of anxiety severity, we constructed separate multivariable linear regression models for male and female patients (Table 4).

**Table 4:** Sex-stratified predictors of anxiety severity (HAMA scores) in *de novo* PD patients.

Variable	Male PD (*n* = 57)	Female PD (*n* = 51)		
	Univariate *p*	Multivariable *p*	Univariate *p*	Multivariable *p*
Demographic variables				
Age	0.123	0.723	0.672	0.592
Clinical variables				
HAMD scores	**<0.001**	**<0.001**	**<0.001**	**<0.001**
PDSS scores	**<0.001**	0.732	**<0.001**	0.517
Modified H-Y stage	**0.041**	**0.013**	**<0.001**	0.436
GMV				
Cingulum_Ant_L	0.378	–	0.362	–
Insula_L	0.526	–	**<0.001**	**0.005**
Insula_R	0.225	–	0.05	–
Precuneus_R	**0.003**	**0.001**	0.403	–

Variables with *p* < 0.05 in univariate linear regression were entered into stepwise multivariable models stratified by sex. *p*-values < 0.05 are in bold. All variance inflation factor (VIF) values were < 2, indicating no multicollinearity.

HAMD, Hamilton Depression Rating Scale; PDSS, Parkinson’s Disease Sleep Scale; H-Y stage, Hoehn and Yahr stage; GMV, Gray matter volume; ACC, Anterior cingulate cortex.

In males, the final stepwise model (adjusted *R*^2^ = 0.876, *p* < 0.001) retained higher HAMD scores (*β* = 0.836, *p* < 0.001), more advanced H-Y stage (*β* = 0.134, *p* = 0.013), and lower right precuneus GMV (*β* = −0.185, *p* = 0.001) as independent predictors. In females, the final model (adjusted *R*^2^ = 0.810, *p* < 0.001) identified higher HAMD scores (*β* = 0.783, *p* < 0.001) and lower left insular GMV (*β* = −0.154, *p* = 0.005) as the only independent predictors.

### 3.8 Sex-specific mediation of GMV in the depression-anxiety relationship

**Figure 4:**
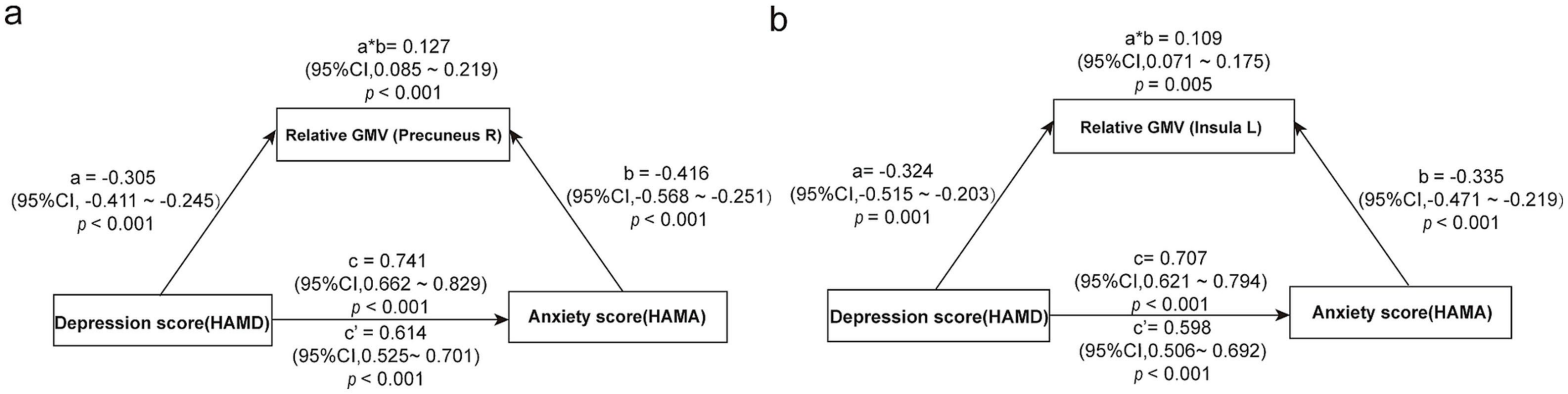
Sex-specific mediation effects of regional gray matter volume on the relationship between depressive and anxiety symptoms in *de novo* PD. **(a)** In male patients, right precuneus GMV partially mediated the relationship between HAMD and HAMA scores (mediation proportion: 17.1%). **(b)** In female patients, left insular GMV partially mediated the same relationship (mediation proportion: 15.4%). Path coefficients are standardized. a, path a; b, path b; a × b, indirect effect; c, total effect; c′, direct effect. GMV, gray matter volume; HAMA, Hamilton Anxiety Rating Scale; HAMD, Hamilton Depression Rating Scale.

Given the strong association between depressive symptoms and anxiety severity identified in the regression models, we performed mediation analyses to examine whether the sex-specific GMV reductions (right precuneus in males, left insula in females) mediated this relationship (Figure 4).

Mediation analyses indicated that right precuneus GMV partially mediated the HAMD–HAMA association in males [indirect effect: 0.127, 95% CI (0.085, 0.219), mediation proportion: 17.1%; Figure 4a]. Similarly, left insular GMV partially mediated this relationship in females [indirect effect: 0.109, 95% CI (0.071, 0.175), mediation proportion: 15.4%; Figure 4b].

## 4 Discussion

### 4.1 Overview of principal findings

Using whole-brain VBM in *de novo* PD patients with strict exclusion of pre-existing primary anxiety disorders, this study revealed sex-specific GMV alterations associated with PD-related anxiety. Correlation analyses confirmed these GMV changes were unrelated to PD motor severity (UPDRS-III) or disease stage (modified H-Y stage), supporting their specificity for PD-related anxiety.

Beyond a shared anxiety-related GMV reduction in the right middle temporal gyrus across sexes, we identified sexually dimorphic neural correlates: anxiety in males was linked to lower right precuneus GMV, while anxiety in females was associated with reduced left insular GMV. These sex-specific GMV alterations partially mediated the relationship between depressive and anxiety symptoms, providing a preliminary neural basis for the frequent co-occurrence of these two non-motor symptoms in PD.

### 4.2 Alignment with existing PD-related anxiety research

Our findings align with and extend prevailing neurocircuitry models of PD-related anxiety, which highlight the critical involvement of the default mode network (DMN), salience network (SN), and limbic system in this non-motor symptom (Cahill et al., 2004; Lopez-Larson et al., 2011; Bangasser and Cuarenta, 2021; Zhao et al., 2025a). Recent preclinical work has further identified that T-type calcium channel-regulated dopaminergic burst firing is a key cellular mechanism underlying these circuit abnormalities in PD-related anxiety (Zhao et al., 2025b). Specifically, we identified the precuneus—a key node of the DMN (Dadario and Sughrue, 2023)—in males, and the insula—a hub of the SN (Uddin, 2015)—in females, suggesting that structural integrity within these large-scale networks regulates anxiety susceptibility in a sex-dependent manner. The common involvement of the right middle temporal gyrus across sexes points to a shared cognitive-affective component of PD-related anxiety, possibly related to social–emotional integration.

In male patients, the association between anxiety and reduced precuneus GMV underscores the role of DMN dysfunction in male-specific anxiety expression. The precuneus is central to self-referential processing and introspective thought (Dadario and Sughrue, 2023), and its structural compromise may predispose individuals to maladaptive self-focused cognition—a core feature of anxiety (Jurado-Coronel et al., 2018; Nicoletti et al., 2023). This is consistent with evidence highlighting the precuneus as a key vulnerability site for neuropsychiatric symptoms in males (Lai, 2018; Billette et al., 2022). Further supporting this sex-specific pattern, previous population-based neuroimaging studies have shown that gray matter structure in the precuneus is modulated by androgen receptor (AR) gene polymorphisms, with significant sex-specific effects (Tan et al., 2020), providing a potential biological basis for the male-specific association between precuneus GMV and anxiety observed in our study.

In our study, the female-specific association between anxiety and reduced GMV in the left insula highlights the prominent role of the SN in anxiety pathogenesis among female PD patients. The anterior insula is a core hub for interoceptive awareness, threat detection, and autonomic emotional regulation (Molnar-Szakacs and Uddin, 2022), and preclinical studies have confirmed that stress-induced alterations in anterior insular activity can drive sex-specific anxiety-related behavioral changes (Shi et al., 2023). Structural GMV reductions in this region may disrupt somatic-emotional integration, thereby contributing to the somatic symptoms of anxiety that are more prevalent in female PD patients (Nagai et al., 2007; Uddin, 2015; Namkung et al., 2017; Seeley, 2019). Notably, the insular cortex has a high density of estrogen receptors (ESR1 and ESR2), and estrogenic signaling has been shown to directly regulate the structural plasticity of insular cortex neurons (Nguyen et al., 2025). Sex steroid receptor gene polymorphisms have also been linked to individual differences in insular gray matter structure in the general population (Tan et al., 2020), offering a well-supported biological mechanism for the female-specific association between insular GMV and anxiety observed in our PD cohort.

These neuroanatomical distinctions may correspond to differing clinical profiles of anxiety between male and female PD patients. The independent association between anxiety severity and disease stage (H-Y stage) in males suggests their anxiety may be more closely tied to global neurodegenerative progression, with the DMN hub (precuneus) being particularly susceptible to accumulating pathology (van der Velden et al., 2018; Pontone et al., 2019; Calipari, 2020). In females, the strong link with insular integrity implies a more direct involvement of non-dopaminergic systems—such as serotonergic or neuroendocrine pathways—in anxiety pathogenesis (Pilotto et al., 2019; Schmidt et al., 2020; Coffeen et al., 2024).

### 4.3 Mediating mechanisms of depression-anxiety co-occurrence

Furthermore, our mediation analysis provides a preliminary neural hypothesis for the frequent co-occurrence of depression and anxiety in PD (Rutten et al., 2017; Upneja et al., 2021). The finding that precuneus GMV partially mediates the depression-anxiety relationship in males, while insular GMV does so in females, suggests that these symptoms may share an upstream neural vulnerability that is expressed through sexually dimorphic networks.

Specifically, depressive symptoms may exacerbate structural deficits in these sex-specific hub regions (precuneus in males, insula in females), thereby precipitating or amplifying anxiety symptoms in PD patients. This underscores that sex differences may manifest not only in static neural correlates of anxiety, but also in the dynamic interplay between affective symptoms in PD (Pallier et al., 2022; Ugursu et al., 2024; Miller et al., 2025).

### 4.4 Translational implications and clinical relevance

From a translational perspective, these results advocate for a sex-informed approach in both research and clinical management of PD-related anxiety. The sex-specific GMV patterns identified in this study may serve as potential imaging biomarkers for differentiating PD-related anxiety from primary anxiety disorders, and for stratifying patients in clinical trials of anti-anxiety interventions.

Future clinical interventions—such as targeted neuromodulation (Koch et al., 2025; Mao et al., 2025) or psychotherapy (Sezer et al., 2022; Gasión et al., 2023)—could consider focusing on the DMN (particularly the precuneus) in male PD patients and the SN (particularly the insula) in female PD patients, to develop personalized treatment strategies. In addition, GMV of the right precuneus and left insula could be used as objective indicators to evaluate the efficacy of anti-anxiety treatment in future longitudinal and interventional studies. However, such clinical applications remain speculative and require further validation through prospective, controlled trials.

#### 4.4.1 Limitations

This study has several limitations that must be acknowledged when interpreting the results. First, the absence of a healthy control group limits our ability to exclude normative sex-related neuroanatomical differences in the general population, and thus we cannot fully disentangle PD-related effects from baseline sex differences in healthy people. Accordingly, all core conclusions of this study are strictly limited to the *de novo* PD patient population, and cannot be extrapolated to comparisons between PD patients and healthy populations. Second, the cross-sectional design of the study precludes causal inferences regarding the relationship between GMV alterations and the development of anxiety in PD. Third, the lack of gonadal hormone and genetic data hinders further exploration of the biological mechanisms underlying the observed sex differences in neural correlates of anxiety. Fourth, the single-center design and relatively small sample size of the subgroup analyses may restrict the generalizability of our findings.

## 5 Conclusion

In summary, this VBM study demonstrates that anxiety in *de novo* PD is associated with sex-specific GMV alterations, characterized by precuneus involvement in males and insula involvement in females. These structural alterations are independent of PD disease severity and partially mediate the co-occurrence between depressive and anxiety symptoms, supporting their specificity as neural correlates of PD-related anxiety. These findings reinforce the importance of biological sex as a critical modulator of the neural substrates of anxiety in PD, and provide a preliminary theoretical basis for the development of sex-informed personalized diagnostic and therapeutic strategies for PD-related anxiety.

## Data Availability

The original contributions presented in the study are included in the article/supplementary material, further inquiries can be directed to the corresponding authors.

## References

[ref1] Ashburner J. (2007). A fast diffeomorphic image registration algorithm. NeuroImage 38, 95–113. doi: 10.1016/j.neuroimage.2007.07.007 17761438

[ref2] Ashburner J. Friston K. J. (2000). Voxel-based morphometry—the methods. NeuroImage 11, 805–821. doi: 10.1006/nimg.2000.0582, 10860804

[ref3] Bangasser D. A. Cuarenta A. (2021). Sex differences in anxiety and depression: circuits and mechanisms. Nat. Rev. Neurosci. 22, 674–684. doi: 10.1038/s41583-021-00513-0, 34545241

[ref4] Billette O. V. Ziegler G. Aruci M. Schütze H. Kizilirmak J. M. Richter A. . (2022). Novelty-related fMRI responses of precuneus and medial temporal regions in individuals at risk for Alzheimer disease. Neurology 99, e775–e788. doi: 10.1212/wnl.0000000000200667, 35995589 PMC9484732

[ref5] Broen M. P. Narayen N. E. Kuijf M. L. Dissanayaka N. N. Leentjens A. F. (2016). Prevalence of anxiety in Parkinson's disease: a systematic review and meta-analysis. Mov. Disord. 31, 1125–1133. doi: 10.1002/mds.26643, 27125963

[ref6] Cahill L. Uncapher M. Kilpatrick L. Alkire M. T. Turner J. (2004). Sex-related hemispheric lateralization of amygdala function in emotionally influenced memory: an FMRI investigation. Learn. Mem. 11, 261–266. doi: 10.1101/lm.70504, 15169855 PMC419728

[ref7] Calipari E. S. (2020). Dopamine release in the midbrain promotes anxiety. Biol. Psychiatry 88, 815–817. doi: 10.1016/j.biopsych.2020.08.016, 33153526 PMC7687288

[ref8] Carey G. Görmezoğlu M. de Jong J. J. A. Hofman P. A. M. Backes W. H. Dujardin K. . (2021). Neuroimaging of anxiety in Parkinson's disease: a systematic review. Mov. Disord. 36, 327–339. doi: 10.1002/mds.28404, 33289195 PMC7984351

[ref9] Cattaneo C. Pagonabarraga J. (2025). Sex differences in Parkinson's disease: a narrative review. Neurol. Ther. 14, 57–70. doi: 10.1007/s40120-024-00687-6, 39630386 PMC11762054

[ref10] Chaudhuri K. R. Pal S. DiMarco A. Whately-Smith C. Bridgman K. Mathew R. . (2002). The Parkinson's disease sleep scale: a new instrument for assessing sleep and nocturnal disability in Parkinson's disease. J. Neurol. Neurosurg. Psychiatry 73, 629–635. doi: 10.1136/jnnp.73.6.629, 12438461 PMC1757333

[ref11] Coffeen U. Ramírez-Rodríguez G. B. Simón-Arceo K. Mercado F. Almanza A. Jaimes O. . (2024). The role of the insular cortex and serotonergic system in the modulation of long-lasting nociception. Cells 13:1718. doi: 10.3390/cells13201718, 39451236 PMC11506361

[ref12] Dadario N. B. Sughrue M. E. (2023). The functional role of the precuneus. Brain 146, 3598–3607. doi: 10.1093/brain/awad181 37254740

[ref13] Douma E. H. de Kloet E. R. (2020). Stress-induced plasticity and functioning of ventral tegmental dopamine neurons. Neurosci. Biobehav. Rev. 108, 48–77. doi: 10.1016/j.neubiorev.2019.10.015, 31666179

[ref14] Erro R. Pappatà S. Amboni M. Vicidomini C. Longo K. Santangelo G. . (2012). Anxiety is associated with striatal dopamine transporter availability in newly diagnosed untreated Parkinson's disease patients. Parkinsonism Relat. Disord. 18, 1034–1038. doi: 10.1016/j.parkreldis.2012.05.022, 22789824

[ref15] Gasión V. Barceló-Soler A. Beltrán-Ruiz M. Hijar-Aguinaga R. Camarero-Grados L. López-Del-Hoyo Y. . (2023). Effectiveness of an amygdala and insula retraining program combined with mindfulness training to improve the quality of life in patients with long COVID: a randomized controlled trial protocol. BMC Complement. Med. Ther. 23:403. doi: 10.1186/s12906-023-04240-0, 37946190 PMC10634181

[ref16] Goetz C. G. Poewe W. Rascol O. Sampaio C. Stebbins G. T. Counsell C. . (2004). Movement disorder society task force report on the Hoehn and Yahr staging scale: status and recommendations. Mov. Disord. 19, 1020–1028. doi: 10.1002/mds.20213, 15372591

[ref17] Goltz F. van der Heide A. Helmich R. C. (2024). Alleviating stress in Parkinson's disease: symptomatic treatment, disease modification, or both? J. Parkinsons Dis. 14, S147–s158. doi: 10.3233/jpd-230211, 38363618 PMC11380242

[ref18] Hamilton M. (1959). The assessment of anxiety states by rating. Br. J. Med. Psychol. 32, 50–55. doi: 10.1111/j.2044-8341.1959.tb00467.x 13638508

[ref19] Hamilton M. (1980). Rating depressive patients. J. Clin. Psychiatry 41, 21–24. 7440521

[ref20] Honey C. J. Kötter R. Breakspear M. Sporns O. (2007). Network structure of cerebral cortex shapes functional connectivity on multiple time scales. Proc. Natl. Acad. Sci. USA 104, 10240–10245. doi: 10.1073/pnas.0701519104, 17548818 PMC1891224

[ref21] Jankovic J. McDermott M. Carter J. Gauthier S. Goetz C. Golbe L. . (1990). Variable expression of Parkinson's disease: a base-line analysis of the DATATOP cohort. The Parkinson Study Group. Neurology 40, 1529–1534. doi: 10.1212/wnl.40.10.1529 2215943

[ref22] Joling M. van den Heuvel O. A. Berendse H. W. Booij J. Vriend C. (2018). Serotonin transporter binding and anxiety symptoms in Parkinson's disease. J. Neurol. Neurosurg. Psychiatry 89, 89–94. doi: 10.1136/jnnp-2017-316193, 28899958

[ref23] Jurado-Coronel J. C. Cabezas R. Ávila Rodríguez M. F. Echeverria V. García-Segura L. M. Barreto G. E. (2018). Sex differences in Parkinson's disease: features on clinical symptoms, treatment outcome, sexual hormones and genetics. Front. Neuroendocrinol. 50, 18–30. doi: 10.1016/j.yfrne.2017.09.002, 28974386

[ref24] Koch G. Casula E. P. Bonnì S. Borghi I. Assogna M. Di Lorenzo F. . (2025). Effects of 52 weeks of precuneus rTMS in Alzheimer's disease patients: a randomized trial. Alzheimer's Res. Ther. 17:69. doi: 10.1186/s13195-025-01709-7, 40176122 PMC11963669

[ref25] Lai C. H. (2018). The regional homogeneity of cingulate-precuneus regions: the putative biomarker for depression and anxiety. J. Affect. Disord. 229, 171–176. doi: 10.1016/j.jad.2017.12.086, 29316519

[ref26] Lee B. Young C. B. Cai W. Yuan R. Ryman S. Kim J. . (2025). Dopaminergic modulation and dosage effects on brain state dynamics and working memory component processes in Parkinson's disease. Nat. Commun. 16:2433. doi: 10.1038/s41467-025-56660-w, 40069154 PMC11897313

[ref27] Leentjens A. F. Dujardin K. Marsh L. Richard I. H. Starkstein S. E. Martinez-Martin P. (2011). Anxiety rating scales in Parkinson's disease: a validation study of the Hamilton anxiety rating scale, the Beck anxiety inventory, and the hospital anxiety and depression scale. Mov. Disord. 26, 407–415. doi: 10.1002/mds.23184, 21384425

[ref28] Li H. Jia J. Yang Z. (2016). Mini-mental state examination in elderly Chinese: a population-based normative study. J. Alzheimer's Dis 53, 487–496. doi: 10.3233/jad-160119, 27163822

[ref29] Liu W. Z. Zhang W. H. Zheng Z. H. Zou J. X. Liu X. X. Huang S. H. . (2020). Identification of a prefrontal cortex-to-amygdala pathway for chronic stress-induced anxiety. Nat. Commun. 11:2221. doi: 10.1038/s41467-020-15920-7, 32376858 PMC7203160

[ref30] Lonka V. Hartikainen S. Tiihonen M. Koponen M. Tolppanen A. M. (2025). The incidence of benzodiazepine and benzodiazepine-related drug use in people with and without Parkinson's disease-a nationwide cohort study. Basic Clin. Pharmacol. Toxicol. 137:e70123. doi: 10.1111/bcpt.70123, 41017167 PMC12477416

[ref31] Lopez-Larson M. P. Anderson J. S. Ferguson M. A. Yurgelun-Todd D. (2011). Local brain connectivity and associations with gender and age. Dev. Cogn. Neurosci. 1, 187–197. doi: 10.1016/j.dcn.2010.10.001, 21516202 PMC3079272

[ref32] Maillet A. Krack P. Lhommée E. Météreau E. Klinger H. Favre E. . (2016). The prominent role of serotonergic degeneration in apathy, anxiety and depression in de novo Parkinson's disease. Brain 139, 2486–2502. doi: 10.1093/brain/aww162, 27538418

[ref33] Mammen J. R. Adams J. L. Mangrum R. Xiao Y. Barbosa W. Tyo M. . (2025). Systematic review and consensus conceptual model of meaningful symptoms and functional impacts in early Parkinson's disease. NPJ Parkinsons Dis. 11:65. doi: 10.1038/s41531-025-00907-2, 40175369 PMC11965473

[ref34] Mao Y. Fan L. Feng C. Dai Z. (2025). Predicting responses of neuromodulation and psychotherapies for major depressive disorder: a coordinate-based meta-analysis of functional magnetic resonance imaging studies. Neurosci. Biobehav. Rev. 172:106120. doi: 10.1016/j.neubiorev.2025.106120 40122358

[ref35] McEwen B. S. Nasca C. Gray J. D. (2016). Stress effects on neuronal structure: Hippocampus, amygdala, and prefrontal cortex. Neuropsychopharmacology 41, 3–23. doi: 10.1038/npp.2015.171, 26076834 PMC4677120

[ref36] McGregor M. M. Nelson A. B. (2019). Circuit mechanisms of Parkinson's disease. Neuron 101, 1042–1056. doi: 10.1016/j.neuron.2019.03.004, 30897356

[ref37] Meoni S. Macerollo A. Moro E. (2020). Sex differences in movement disorders. Nat. Rev. Neurol. 16, 84–96. doi: 10.1038/s41582-019-0294-x 31900464

[ref38] Miller C. N. Li Y. Beier K. T. Aoto J. (2025). Acute stress causes sex-specific changes to ventral subiculum synapses, circuitry, and anxiety-like behavior. Nat. Commun. 16:5604. doi: 10.1038/s41467-025-60512-y, 40595510 PMC12217864

[ref39] Mohebi A. Pettibone J. R. Hamid A. A. Wong J. T. Vinson L. T. Patriarchi T. . (2019). Dissociable dopamine dynamics for learning and motivation. Nature 570, 65–70. doi: 10.1038/s41586-019-1235-y 31118513 PMC6555489

[ref40] Molnar-Szakacs I. Uddin L. Q. (2022). Anterior insula as a gatekeeper of executive control. Neurosci. Biobehav. Rev. 139:104736. doi: 10.1016/j.neubiorev.2022.104736, 35700753

[ref41] Moriyama T. S. Felicio A. C. Chagas M. H. Tardelli V. S. Ferraz H. B. Tumas V. . (2011). Increased dopamine transporter density in Parkinson's disease patients with social anxiety disorder. J. Neurol. Sci. 310, 53–57. doi: 10.1016/j.jns.2011.06.056, 21783205

[ref42] Nagai M. Kishi K. Kato S. (2007). Insular cortex and neuropsychiatric disorders: a review of recent literature. Eur. Psychiatry 22, 387–394. doi: 10.1016/j.eurpsy.2007.02.006, 17416488

[ref43] Nakajima M. Görlich A. Heintz N. (2014). Oxytocin modulates female sociosexual behavior through a specific class of prefrontal cortical interneurons. Cell 159, 295–305. doi: 10.1016/j.cell.2014.09.020, 25303526 PMC4206218

[ref44] Namkung H. Kim S. H. Sawa A. (2017). The insula: an underestimated brain area in clinical neuroscience, psychiatry, and neurology. Trends Neurosci. 40, 200–207. doi: 10.1016/j.tins.2017.02.002, 28314446 PMC5538352

[ref45] Nguyen C. Mondoloni S. Le Borgne T. Centeno I. Come M. Jehl J. . (2021). Nicotine inhibits the VTA-to-amygdala dopamine pathway to promote anxiety. Neuron 109, 2604–2615.e9. doi: 10.1016/j.neuron.2021.06.013, 34242565

[ref46] Nguyen R. Rahyab R. Deshpande A. Legge E. Almeida J. Herz S. M. . (2025). Estrogenic regulation of perineuronal nets in the mouse insular cortex and hippocampus. Neuropharmacology 279:110641. doi: 10.1016/j.neuropharm.2025.110641, 40825495 PMC12415989

[ref47] Nicoletti A. Baschi R. Cicero C. E. Iacono S. Re V. L. Luca A. . (2023). Sex and gender differences in Alzheimer's disease, Parkinson's disease, and amyotrophic lateral sclerosis: a narrative review. Mech. Ageing Dev. 212:111821. doi: 10.1016/j.mad.2023.111821, 37127082

[ref48] Pallier P. N. Ferrara M. Romagnolo F. Ferretti M. T. Soreq H. Cerase A. (2022). Chromosomal and environmental contributions to sex differences in the vulnerability to neurological and neuropsychiatric disorders: implications for therapeutic interventions. Prog. Neurobiol. 219:102353. doi: 10.1016/j.pneurobio.2022.102353 36100191

[ref49] Pilotto A. di Schiano Cola F. Premi E. Grasso R. Turrone R. Gipponi S. . (2019). Extrastriatal dopaminergic and serotonergic pathways in Parkinson's disease and in dementia with Lewy bodies: a (123)I-FP-CIT SPECT study. Eur. J. Nucl. Med. Mol. Imaging 46, 1642–1651. doi: 10.1007/s00259-019-04324-5, 31098748

[ref50] Pontone G. M. Dissanayka N. Apostolova L. Brown R. G. Dobkin R. Dujardin K. . (2019). Report from a multidisciplinary meeting on anxiety as a non-motor manifestation of Parkinson's disease. NPJ Parkinsons Dis. 5:30. doi: 10.1038/s41531-019-0102-8, 31840044 PMC6906437

[ref51] Pontone G. M. Mills K. A. (2021). Optimal treatment of depression and anxiety in Parkinson's disease. Am. J. Geriatr. Psychiatry 29, 530–540. doi: 10.1016/j.jagp.2021.02.037, 33648830

[ref52] Postuma R. B. Berg D. Stern M. Poewe W. Olanow C. W. Oertel W. . (2015). MDS clinical diagnostic criteria for Parkinson's disease. Mov. Disord. 30, 1591–1601. doi: 10.1002/mds.26424, 26474316

[ref53] Rigney N. Whylings J. de Vries G. J. Petrulis A. (2021). Sex differences in the control of social investigation and anxiety by vasopressin cells of the paraventricular nucleus of the hypothalamus. Neuroendocrinology 111, 521–535. doi: 10.1159/000509421, 32541145 PMC7736187

[ref54] Rutten S. Vriend C. van der Werf Y. D. Berendse H. W. Weintraub D. van den Heuvel O. A. (2017). The bidirectional longitudinal relationship between insomnia, depression and anxiety in patients with early-stage, medication-naïve Parkinson's disease. Parkinsonism Relat. Disord. 39, 31–36. doi: 10.1016/j.parkreldis.2017.01.015, 28365203 PMC5441947

[ref55] Schmidt C. Skandali N. Gleesborg C. Kvamme T. L. Schmidt H. Frisch K. . (2020). The role of dopaminergic and serotonergic transmission in the processing of primary and monetary reward. Neuropsychopharmacology 45, 1490–1497. doi: 10.1038/s41386-020-0702-3, 32392573 PMC7360589

[ref56] Seeley W. W. (2019). The salience network: a neural system for perceiving and responding to homeostatic demands. J. Neurosci. 39, 9878–9882. doi: 10.1523/jneurosci.1138-17.2019, 31676604 PMC6978945

[ref57] Sezer I. Pizzagalli D. A. Sacchet M. D. (2022). Resting-state fMRI functional connectivity and mindfulness in clinical and non-clinical contexts: a review and synthesis. Neurosci. Biobehav. Rev. 135:104583. doi: 10.1016/j.neubiorev.2022.104583, 35202647 PMC9083081

[ref58] Shi T. Feng S. Zhou Z. Li F. Fu Y. Zhou W. (2023). Stress-altering anterior insular cortex activity affects risk decision-making behavior in mice of different sexes. Front. Cell. Neurosci. 17:1094808. doi: 10.3389/fncel.2023.1094808, 36761354 PMC9902351

[ref59] Tan G. C. Chu C. Lee Y. T. Tan C. C. K. Ashburner J. Wood N. W. . (2020). The influence of microsatellite polymorphisms in sex steroid receptor genes ESR1, ESR2 and AR on sex differences in brain structure. NeuroImage 221:117087. doi: 10.1016/j.neuroimage.2020.117087, 32593802 PMC8960998

[ref60] Thobois S. Prange S. Sgambato-Faure V. Tremblay L. Broussolle E. (2017). Imaging the etiology of apathy, anxiety, and depression in Parkinson's disease: implication for treatment. Curr. Neurol. Neurosci. Rep. 17:76. doi: 10.1007/s11910-017-0788-0 28822071

[ref61] Tropea T. F. Hartstone W. Amari N. Baum D. Rick J. Suh E. . (2024). Genetic and phenotypic characterization of Parkinson's disease at the clinic-wide level. NPJ Parkinsons Dis. 10:97. doi: 10.1038/s41531-024-00690-6, 38702337 PMC11068880

[ref62] Uddin L. Q. (2015). Salience processing and insular cortical function and dysfunction. Nat. Rev. Neurosci. 16, 55–61. doi: 10.1038/nrn3857, 25406711

[ref63] Ugursu B. Sah A. Sartori S. Popp O. Mertins P. Dunay I. R. . (2024). Microglial sex differences in innate high anxiety and modulatory effects of minocycline. Brain Behav. Immun. 119, 465–481. doi: 10.1016/j.bbi.2024.03.035, 38552926

[ref64] Upneja A. Paul B. S. Jain D. Choudhary R. Paul G. (2021). Anxiety in Parkinson's disease: correlation with depression and quality of life. J. Neurosci. Rural Pract. 12, 323–328. doi: 10.1055/s-0041-1722840, 33986584 PMC8110433

[ref65] van der Velden R. M. J. Broen M. P. G. Kuijf M. L. Leentjens A. F. G. (2018). Frequency of mood and anxiety fluctuations in Parkinson's disease patients with motor fluctuations: a systematic review. Mov. Disord. 33, 1521–1527. doi: 10.1002/mds.27465, 30225905

[ref66] van Wegen E. E. H. van Balkom T. D. Hirsch M. A. Rutten S. van den Heuvel O. A. (2024). Non-pharmacological interventions for depression and anxiety in Parkinson's disease. J. Parkinsons Dis. 14, S135–s146. doi: 10.3233/jpd-230228, 38607762 PMC11380297

[ref67] Vanasse T. J. Fox P. M. Barron D. S. Robertson M. Eickhoff S. B. Lancaster J. L. . (2018). BrainMap VBM: an environment for structural meta-analysis. Hum. Brain Mapp. 39, 3308–3325. doi: 10.1002/hbm.24078, 29717540 PMC6866579

[ref68] Volkmann J. Daniels C. Witt K. (2010). Neuropsychiatric effects of subthalamic neurostimulation in Parkinson disease. Nat. Rev. Neurol. 6, 487–498. doi: 10.1038/nrneurol.2010.111, 20680036

[ref69] Vriend C. Boedhoe P. S. Rutten S. Berendse H. W. van der Werf Y. D. van den Heuvel O. A. (2016). A smaller amygdala is associated with anxiety in Parkinson's disease: a combined FreeSurfer-VBM study. J. Neurol. Neurosurg. Psychiatry 87, 493–500. doi: 10.1136/jnnp-2015-310383, 25986365

[ref70] Wang J. Sun L. Chen L. Sun J. Xie Y. Tian D. . (2023). Common and distinct roles of amygdala subregional functional connectivity in non-motor symptoms of Parkinson's disease. NPJ Parkinsons Dis. 9:28. doi: 10.1038/s41531-023-00469-1, 36806219 PMC9938150

[ref71] Wang L. Xiong X. Liu J. Liu R. Liao J. Li F. . (2025). Gray matter structural and functional brain abnormalities in Parkinson's disease: a meta-analysis of VBM and ALFF data. J. Neurol. 272:276. doi: 10.1007/s00415-025-12934-3, 40106017

[ref72] Wang X. Zhang J. Yuan Y. Li T. Zhang L. Ding J. . (2017). Cerebral metabolic change in Parkinson's disease patients with anxiety: a FDG-PET study. Neurosci. Lett. 653, 202–207. doi: 10.1016/j.neulet.2017.05.062, 28579485

[ref73] Weintraub D. Newberg A. B. Cary M. S. Siderowf A. D. Moberg P. J. Kleiner-Fisman G. . (2005). Striatal dopamine transporter imaging correlates with anxiety and depression symptoms in Parkinson's disease. J. Nucl. Med. 46, 227–232. 15695780

[ref74] Xu X. Han Q. Lin J. Wang L. Wu F. Shang H. (2020). Grey matter abnormalities in Parkinson's disease: a voxel-wise meta-analysis. Eur. J. Neurol. 27, 653–659. doi: 10.1111/ene.14132, 31770481

[ref75] Zhang P. Zhang Y. Luo Y. Wang L. Wang K. (2022). Regional activity alterations in Parkinson's disease patients with anxiety disorders: a resting-state functional magnetic resonance imaging study. Front. Aging Neurosci. 14:1055160. doi: 10.3389/fnagi.2022.1055160, 36589538 PMC9800784

[ref76] Zhao J. Jia H. Ma P. Zhu D. Fang Y. (2025a). Multidimensional mechanisms of anxiety and depression in Parkinson's disease: integrating neuroimaging, neurocircuits, and molecular pathways. Pharmacol. Res. 215:107717. doi: 10.1016/j.phrs.2025.107717, 40157405

[ref77] Zhao J. Zhu D. Chen Y. Ma P. Li S. Ye S. . (2025b). T-type calcium channels attenuate anxiety in MPTP-treated mice through modulating burst firing of dopaminergic neuron. Neuropharmacology 272:110424. doi: 10.1016/j.neuropharm.2025.110424, 40118209

